# Correction to “Shiga‐Like Toxin I Exerts Specific and Potent Anti‐Tumour Efficacy Against Gastric Cancer Cell Proliferation When Driven by Tumour‐Preferential Frizzled‐7 Promoter”

**DOI:** 10.1111/cpr.70030

**Published:** 2025-04-01

H. Xu, L. Peng, M. Shen, Y. Xia, Z. Li, and N. He, “Shiga‐Like Toxin I Exerts Specific and Potent Anti‐Tumour Efficacy Against Gastric Cancer Cell Proliferation When Driven by Tumour‐Preferential Frizzled‐7 Promoter,” *Cell Proliferation* 52, no. 3 (2019): e12607, https://doi.org/10.1111/cpr.12607.

Figure 1A consists of three flow cytometry graphs. During the process of splicing, due to opening multiple pictures, a non‐SGC7901 cell graph was accidentally selected. We reproduce Figure 1 with the correct flow cytometry graphs in Figure 1A below.

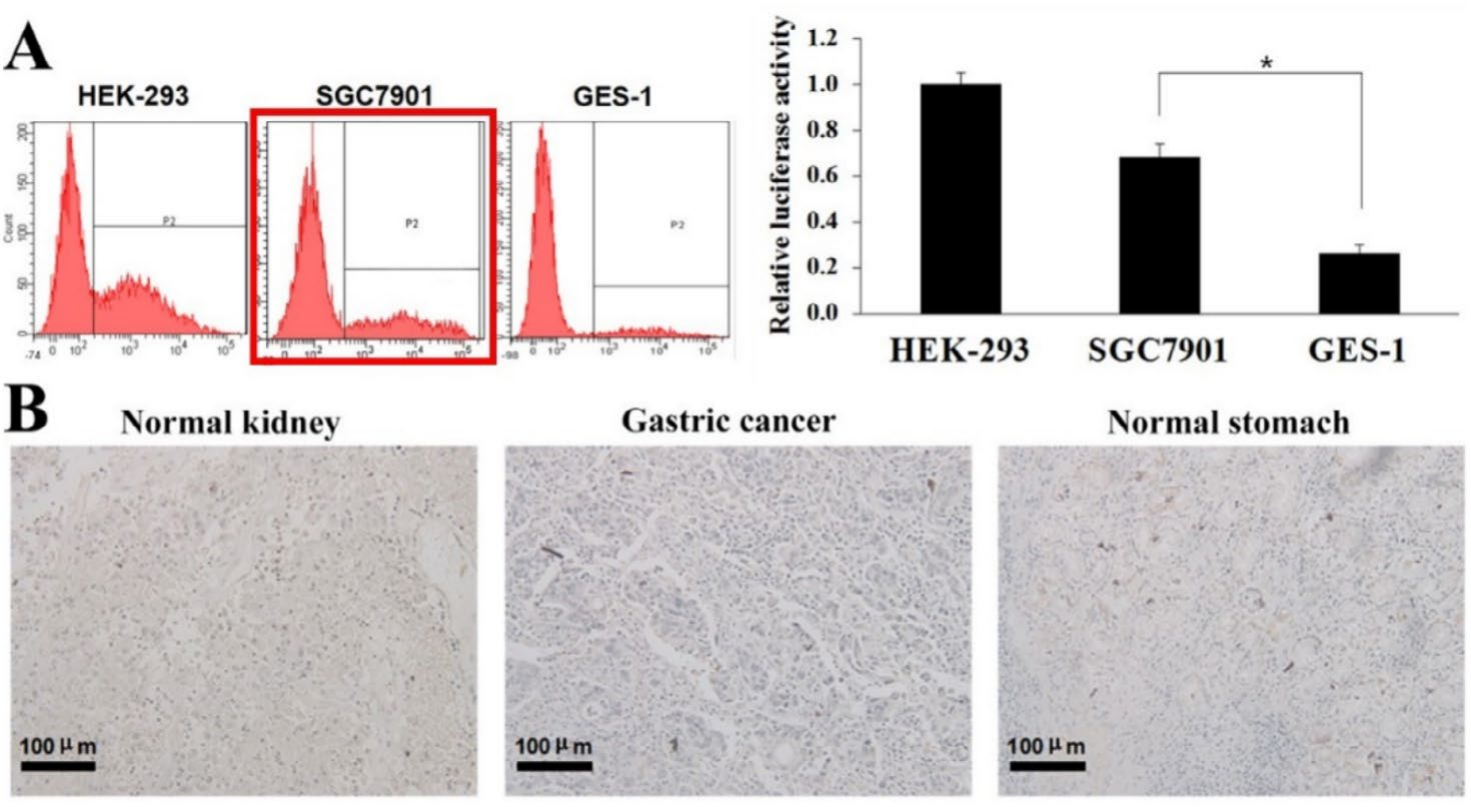

Figure 3B: Due to the large number of photographs and their similar appearances, the light microscope photograph of SGC7901 cell's control group and the electron microscopy photographs (GES‐1 cell: pFZD7 group and pFZD7‐Stx1 group, SGC7901 cell: pFZD7 group) were mistakenly selected. We reproduce Figure 3 with the correct microscopy photographs in Figure 3B below.

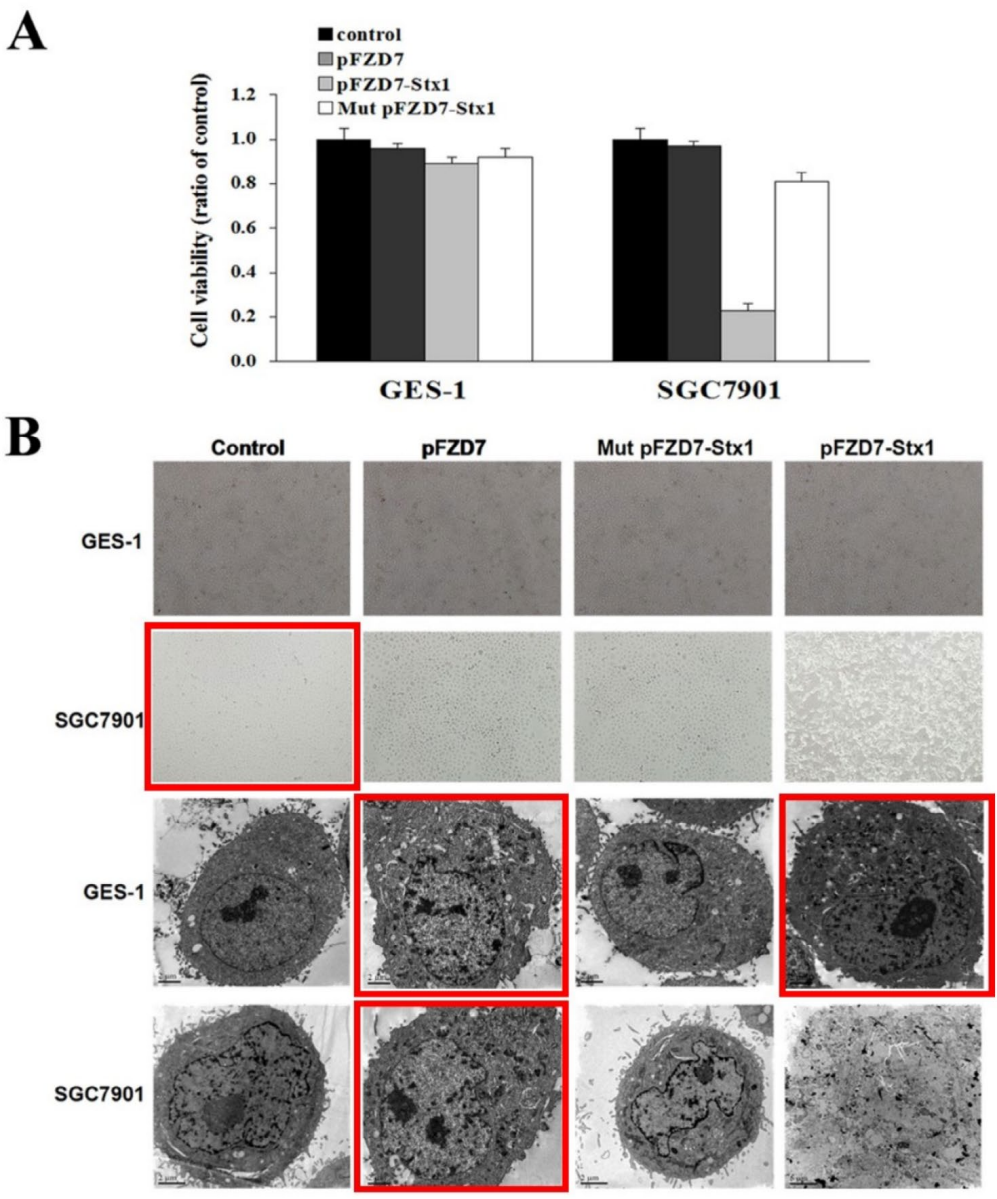

Figure 4B: A large number of agarose gel electrophoresis experiments were conducted during the draft stage of the paper, and the experimental data in the paper were modified. However, the images were not updated at the time of final submission, resulting in the use of incorrect pictures. We reproduce Figure 4 with the updated agarose gel electrophoresis image in Figure 4B below.

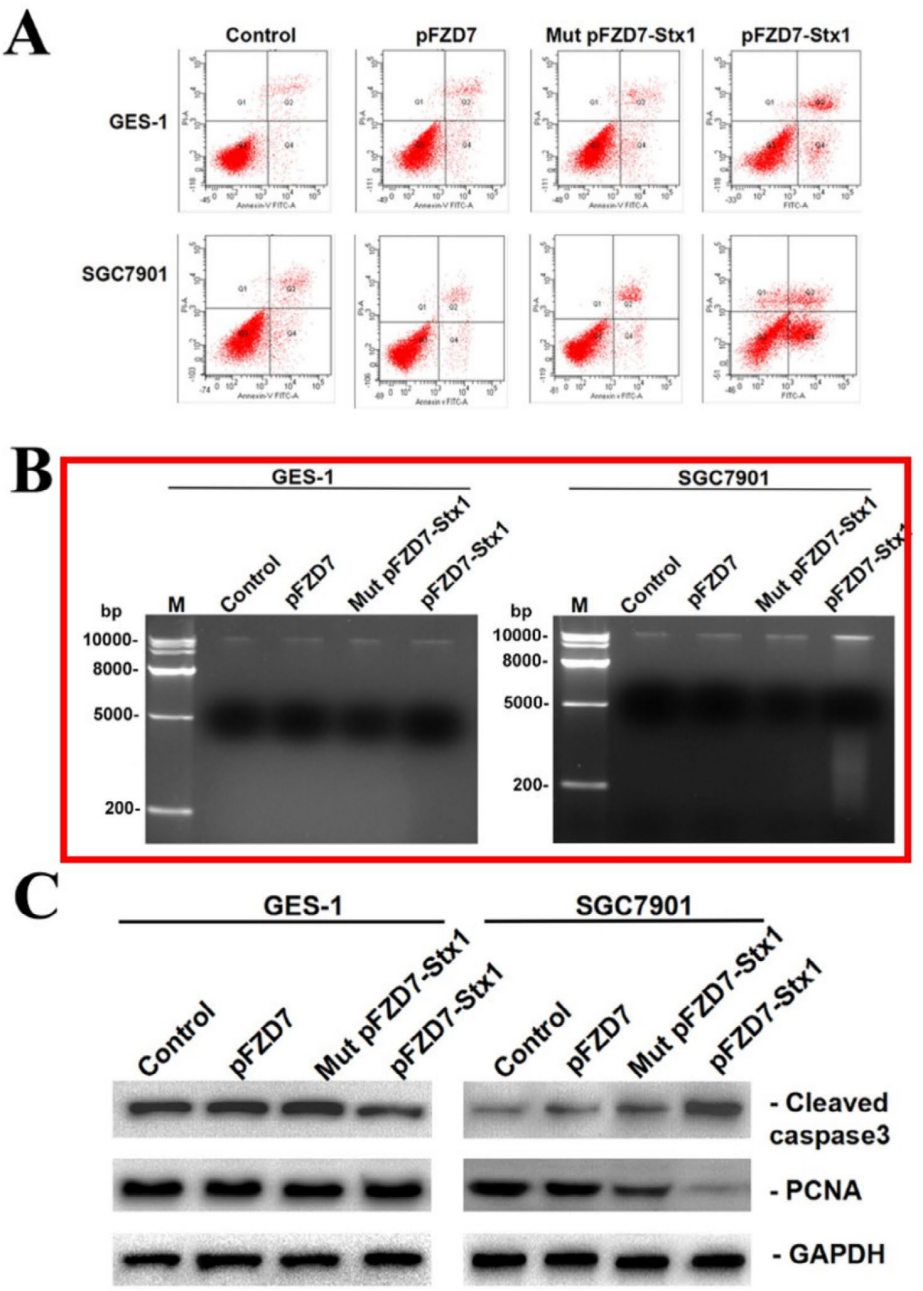

Figure 5A: When editing the image, multiple mouse photographs were combined into one figure. Due to the large number of photographs and their similar appearances, the third photograph of the Mut pFZD7‐Stx1 group was mistakenly selected as the third photograph of the pFZD7 group, resulting in a duplicate image. We reproduce Figure 5, replacing the mouse photograph with that of pFZD7 group mouse in Figure 5A, below.

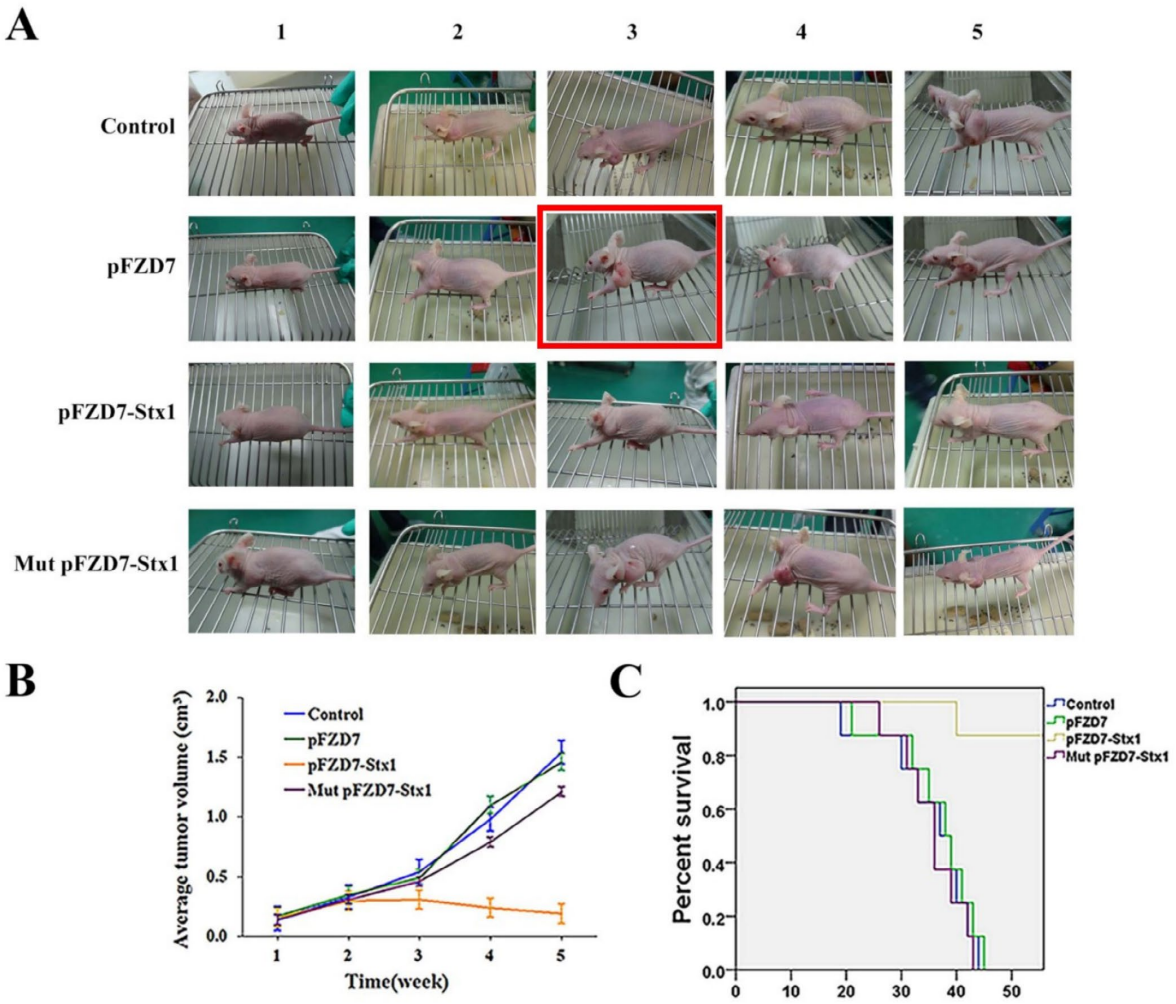

We apologize for these errors.

